# Supplementary figures and images for: Evolution of highly pathogenic H5N1 influenza A virus in the central nervous system of ferrets
Source: PLoS Pathog. 2023 Mar 10;19(3):e1011214. doi: 10.1371/journal.ppat.1011214 (PMC10032531; doi:10.1371/journal.ppat.1011214)

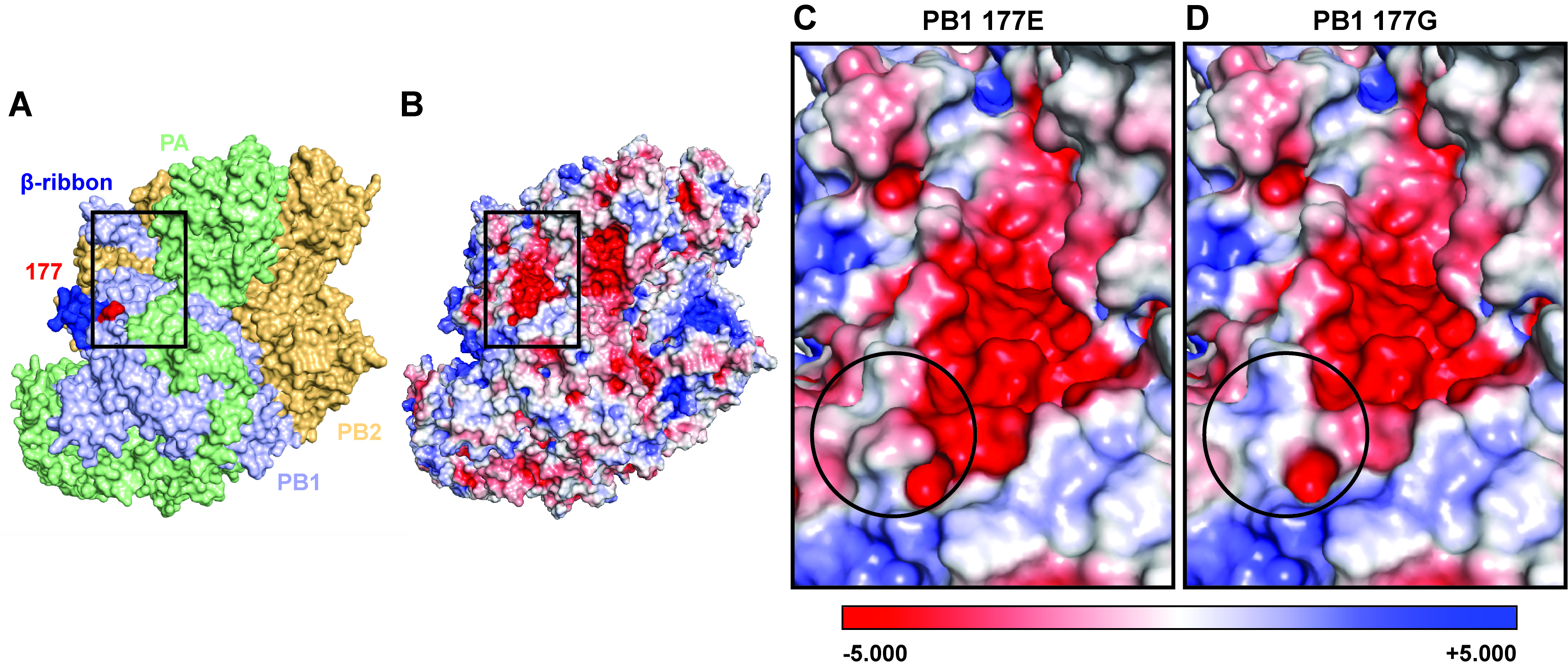

Supplement: S1 Fig — Overview of the electrostatic surface potential of the polymerase complex (A) with closeup view of the PB1 at residue 177 with a glutamic acid (B) and glycine (C). Colors indicate a negative potential (red) and positive potential (blue) according to the color-coded electrostatic surface (unit KT/e). (TIF) [file ppat.1011214.s001.tif]

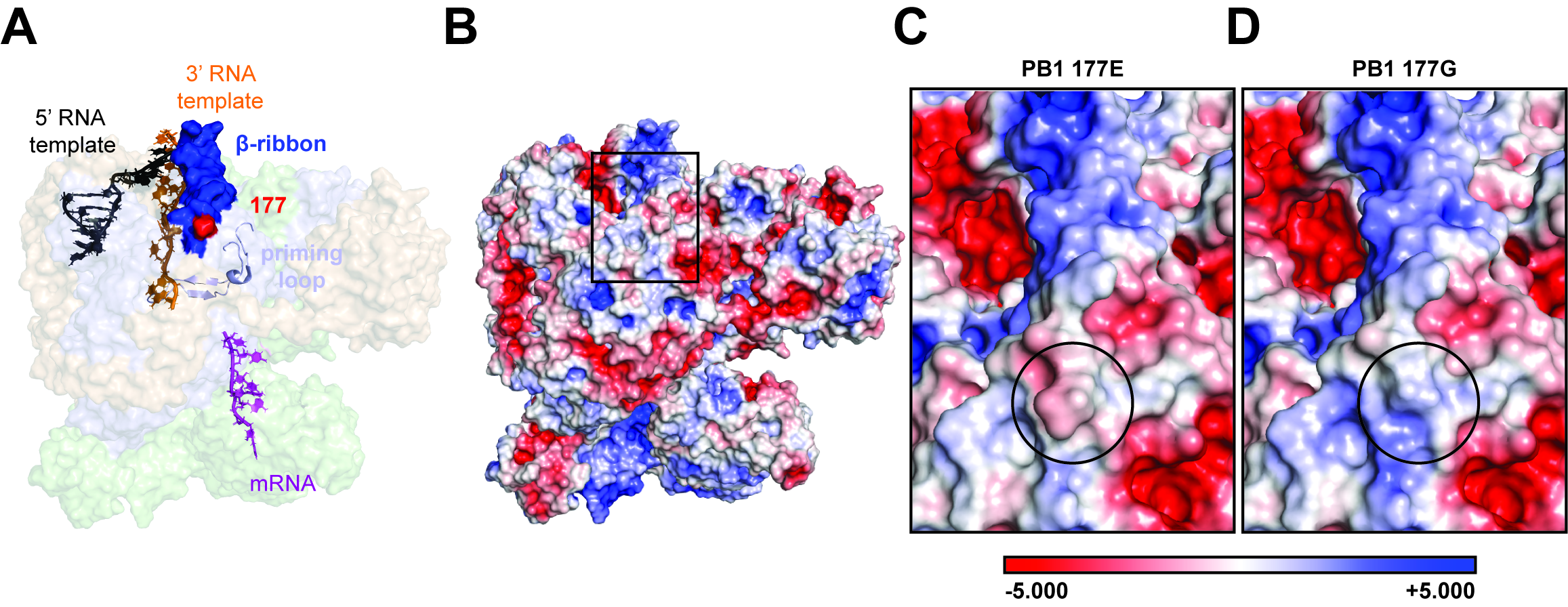

Supplement: S2 Fig — Electrostatic surface potential of the polymerase and in proximity of PB1 residue 177 with bound vRNA promotor (A). Overview of the electrostatic surface potential of the polymerase complex (B) with closeup view of the PB1 at residue 177 with a glutamic acid (C) and glycine (D). Colors indicate a negative potential (red) and positive potential (blue) according to the color scale bar. (TIF) [file ppat.1011214.s002.tif]

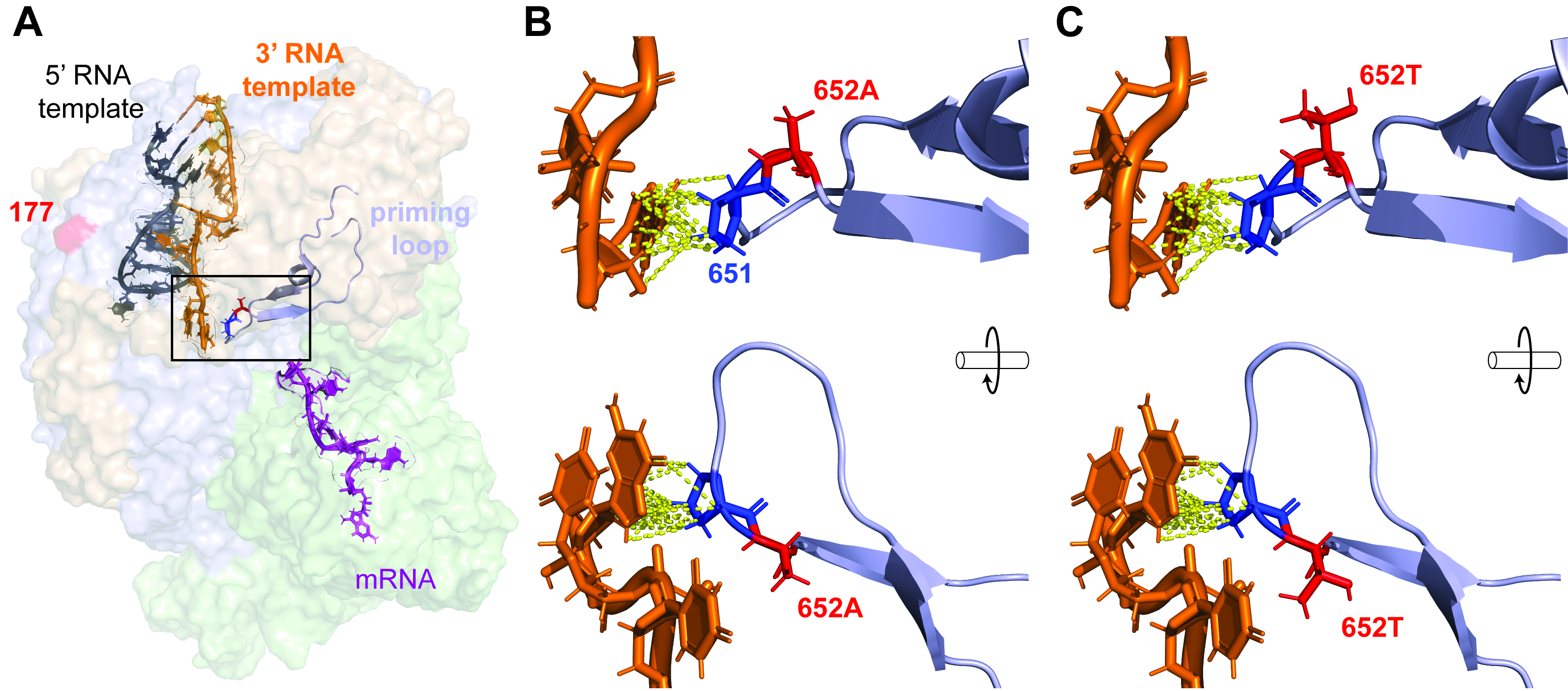

Supplement: S3 Fig — (A), overview of the polymerase subdomains with PB1 (light-blue), PB2 (pale-yellow), and PA (pale-green), 5’ RNA template, 3’ RNA template, and mRNA product. (B), location of PB1 residue 651 (blue) and 652 (red) within the priming loop (light-blue). (C), location of PB1 residue 651 (blue) in contact (yellow dotted line) with the 3’ RNA template. (TIF) [file ppat.1011214.s003.tif]

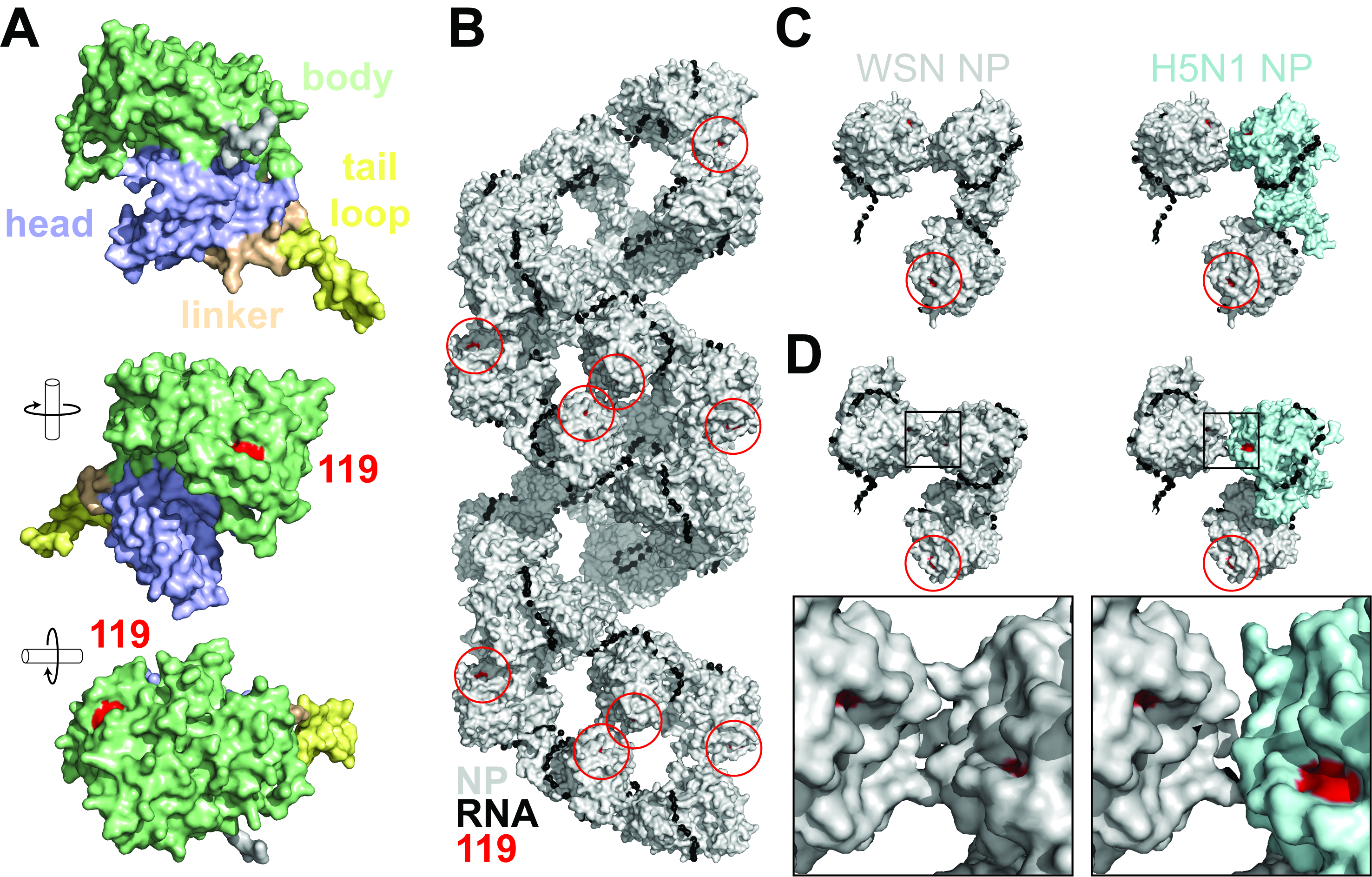

Supplement: S4 Fig — (A), overview of the vRNP complex with NP (grey), RNA (black) and residue 119 (red). (B), the H5N1 NP (light-blue) superimposed on WSN NP (grey) with residue 119 (red) and RNA (black). (C), same as B, alternate view with closeup view displaying NP-interactions on opposite strands. (TIF) [file ppat.1011214.s004.tif]

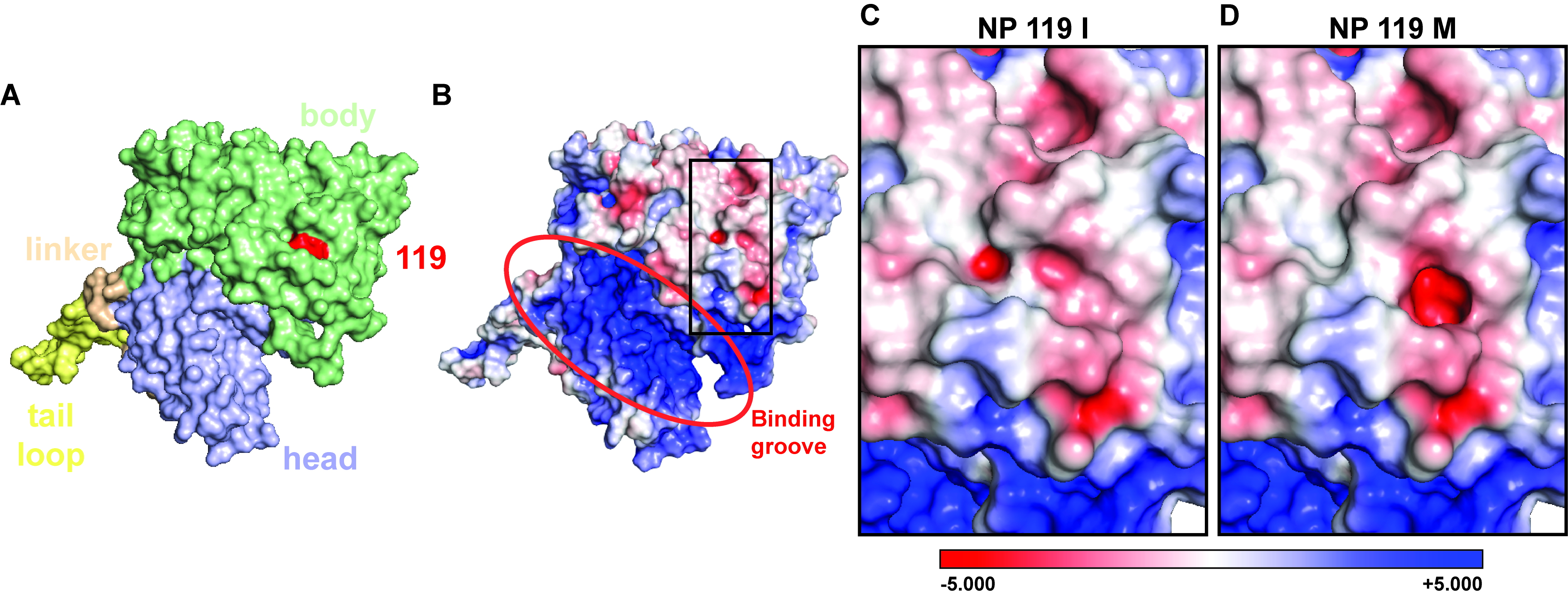

Supplement: S5 Fig — Overview of the electrostatic surface potential of the polymerase complex (B) with closeup view (C & D) of the NP at residue 119 with an isoleucine (I) and methionine (M). Colors indicate a negative potential (red) and positive potential (blue) according to the color scale bar. (TIF) [file ppat.1011214.s005.tif]

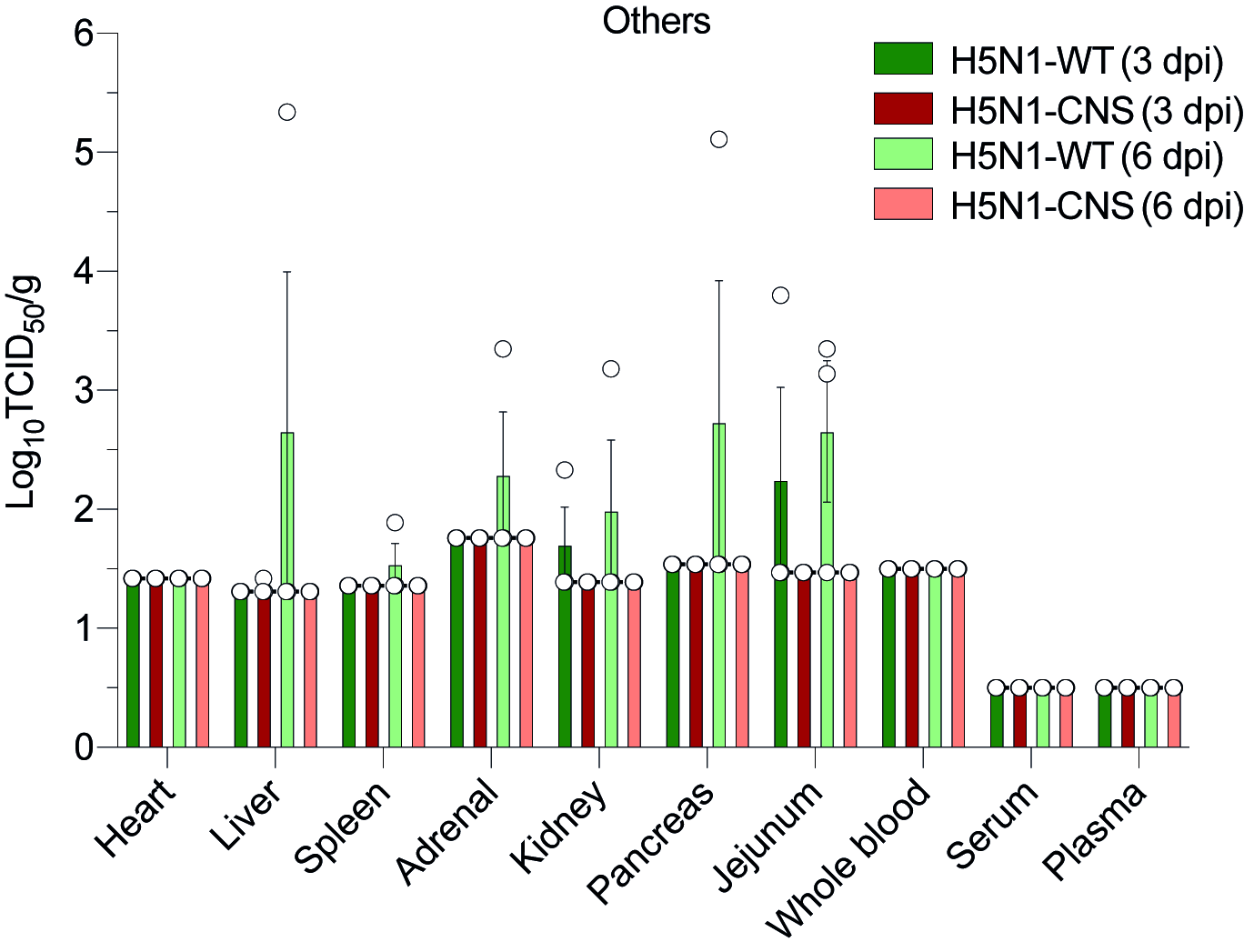

Supplement: S6 Fig — Dots represent individual ferrets while bars and lines represent means ±SDs respectively. Statistical analysis was performed using multiple independent unpaired t-tests. TCID; tissue culture infectious dose. Dotted lines represent the limit of detection. (TIF) [file ppat.1011214.s006.tif]
