# Supplementary material for: Evolution of highly pathogenic H5N1 influenza A virus in the central nervous system of ferrets
Source: PLoS Pathog. 2023 Mar 10;19(3):e1011214. doi: 10.1371/journal.ppat.1011214 (PMC10032531; doi:10.1371/journal.ppat.1011214)
Supplement: S1 Table — (DOCX) [file ppat.1011214.s009.docx]

**S1 Table. In vivo stability of molecular signatures for CNS-mutations’ sites at 3 dpi and alternative consensus alleles.**

| Gene segment | PB1 | |  | PA |  | NP |
| --- | --- | --- | --- | --- | --- | --- |
| Amino acid # | 177 | 652 |  | 404 |  | 119 |
| Reference | E | A |  | A |  | I |
| Nasal turbinates |  |  |  |  |  |  |
| F1 | E | A |  | A |  | I |
| F2 | E | A |  | A |  | I |
| F3 | E | A |  | A |  | I |
| Olfactory bulb |  |  |  |  |  |  |
| F1 | E | A |  | A |  | I |
| F2 | E | A |  | T |  | I |
| F3 | E | A |  | A |  | I |
| Nasal turbinates |  |  |  |  |  |  |
| F4 | G | T |  | A |  | M |
| F5 | G | T |  | A |  | M |
| F6 | G | T |  | A |  | M |
| Olfactory bulb |  |  |  |  |  |  |
| F4 | G | T |  | A |  | M |
| F5 | G | T |  | A |  | M |
| F6 | G | T |  | A |  | M |
