# Supplementary material for: Evolution of highly pathogenic H5N1 influenza A virus in the central nervous system of ferrets
Source: PLoS Pathog. 2023 Mar 10;19(3):e1011214. doi: 10.1371/journal.ppat.1011214 (PMC10032531; doi:10.1371/journal.ppat.1011214)
Supplement: S2 Table — (DOCX) [file ppat.1011214.s010.docx]

**S2 Table. In vivo stability of molecular signatures for CNS-mutations’ sites at 6 dpi and alternative consensus alleles.**

| Gene segement | PB2 | |  | PB1 | | | | |  | HA |  | NP | |  | M (M1) |  | M (M2) |
| --- | --- | --- | --- | --- | --- | --- | --- | --- | --- | --- | --- | --- | --- | --- | --- | --- | --- |
| Amino acid | 178 | 281 |  | 38 | 177 | 262 | 520 | 652 |  | 452 |  | 119 | 398 |  | 229 |  | 16 |
| Reference | T | L |  | Y | E | I | S | A |  | R |  | I | Q |  | L |  | E |
| Nasal turbinates |  |  |  |  |  |  |  |  |  |  |  |  |  |  |  |  |  |
| F7 | T | L |  | Y | E | I | S | A |  | R |  | I | Q |  | L |  | E |
| F8 | T | L |  | Y | E | I | S | A |  | R |  | I | Q |  | L |  | E |
| F9 | T | L |  | Y | E | I | S | A |  | R |  | I | Q |  | L |  | E |
| Olfactory bulb |  |  |  |  |  |  |  |  |  |  |  |  |  |  |  |  |  |
| F7 | A | L |  | Y | E | I | S | A |  | K |  | I | Q |  | L |  | G |
| F8 | T | L |  | Y | E | I | S | A |  | R |  | I | Q |  | L |  | E |
| F9 | T | L |  | Y | E | I | S | A |  | R |  | I | Q |  | L |  | E |
| Cerebrum |  |  |  |  |  |  |  |  |  |  |  |  |  |  |  |  |  |
| F7 | T | L |  | Y | E | V | S | A |  | R |  | I | Q |  | L |  | E |
| F8 | T | L |  | Y | E | I | S | A |  | R |  | I | Q |  | L |  | E |
| F9 | T | L |  | H | E | I | S | A |  | R |  | I | Q |  | L |  | E |
| Cerebellum |  |  |  |  |  |  |  |  |  |  |  |  |  |  |  |  |  |
| F7 | T | L |  | Y | E | V | S | A |  | R |  | I | Q |  | L |  | E |
| F8 | T | L |  | Y | E | I | S | A |  | R |  | I | Q |  | L |  | E |
| F9 | T | L |  | H | E | I | S | A |  | R |  | I | Q |  | L |  | E |
| Brainstem |  |  |  |  |  |  |  |  |  |  |  |  |  |  |  |  |  |
| F7 | T | L |  | Y | E | V | S | A |  | R |  | I | Q |  | L |  | E |
| F8 | T | L |  | Y | E | I | S | A |  | R |  | I | Q |  | L |  | E |
| F9 | T | L |  | Y | E | I | S | A |  | R |  | I | Q |  | L |  | E |
| CSF |  |  |  |  |  |  |  |  |  |  |  |  |  |  |  |  |  |
| F7 | T | L |  | Y | E | V | S | A |  | R |  | I | Q |  | L |  | E |
| F9 | T | L |  | H | E | I | S | A |  | R |  | I | Q |  | L |  | E |
| Extra |  |  |  |  |  |  |  |  |  |  |  |  |  |  |  |  |  |
| F7 (jejunum) | T | P |  | Y | E | I | S | A |  | R |  | I | Q |  | L |  | E |
|  |  |  |  |  |  |  |  |  |  |  |  |  |  |  |  |  |  |
| Nasal turbinates |  |  |  |  |  |  |  |  |  |  |  |  |  |  |  |  |  |
| F10 | T | L |  | Y | G | I | S | T |  | R |  | M | Q |  | L |  | E |
| F11 | T | L |  | Y | G | I | S | T |  | R |  | M | Q |  | L |  | E |
| F12 | T | L |  | Y | G | I | S | T |  | R |  | M | Q |  | L |  | E |
| Olfactory bulb |  |  |  |  |  |  |  |  |  |  |  |  |  |  |  |  |  |
| F10 | T | L |  | Y | G | I | S | T |  | R |  | M | Q |  | L |  | E |
| F11 | T | L |  | Y | G | I | S | T |  | R |  | M | Q |  | L |  | E |
| F12 | T | L |  | Y | G | I | S | T |  | R |  | M | Q |  | L |  | E |
| Cerebrum |  |  |  |  |  |  |  |  |  |  |  |  |  |  |  |  |  |
| F10 | T | L |  | Y | G | I | S | T |  | R |  | M | R |  | L |  | E |
| F11 |  |  |  |  |  |  |  |  |  |  |  |  |  |  | M |  | E |
| F12 | T | L |  | Y | G | I | S | T |  | R |  | M | Q |  | L |  | E |
| Cerebellum |  |  |  |  |  |  |  |  |  |  |  |  |  |  |  |  |  |
| F10 | T | L |  | Y | G | I | S | T |  | R |  | M | R |  | L |  | E |
| F12 | T | L |  | Y | G | I | S | T |  | R |  | M | Q |  | L |  | E |
| Brainstem |  |  |  |  |  |  |  |  |  |  |  |  |  |  |  |  |  |
| F10 | T | L |  | Y | G | I | S | T |  | R |  | M | R |  | L |  | E |
| F11 |  |  |  | Y | G | I | P | T |  | R |  | M | Q |  | L |  |  |
| F12 | T | L |  | Y | G | I | S | T |  | R |  | M | Q |  | L |  | E |
| CSF |  |  |  |  |  |  |  |  |  |  |  |  |  |  |  |  |  |
| F10 | T | L |  | Y | G | I | S | T |  | R |  | M | R |  | L |  | E |
| F12 | T | L |  | Y | G | I | S | T |  | R |  | M | Q |  | L |  | E |
| Extra |  |  |  |  |  |  |  |  |  |  |  |  |  |  |  |  |  |
| F10 (lung) | T | L |  | Y | G | I | S | T |  | R |  | M | Q |  | L |  | E |
