# Supplementary material for: Evolution of highly pathogenic H5N1 influenza A virus in the central nervous system of ferrets
Source: PLoS Pathog. 2023 Mar 10;19(3):e1011214. doi: 10.1371/journal.ppat.1011214 (PMC10032531; doi:10.1371/journal.ppat.1011214)
Supplement: Attachment — Submitted filename: Rebuttal letter.docx [file ppat.1011214.s016.docx]

**Rebuttal PATHOGENS-D-22-01166**

We have addressed all reviewers comments below in blue.

Reviewer's Responses to Questions

**Part I – Summary**

Please use this section to discuss strengths/weaknesses of study, novelty/significance, general execution and scholarship.

Reviewer #1: Siegers et al have performed detailed experiments to explore the mechanisms by which the HPAI H5N1 virus evolves in the mammalian CNS. The authors demonstrate that an HPAI H5N1 virus can enter the CNS via the olfactory nerve without a genetic bottleneck. And the authors conclude that viral dynamics within the CNS are conducive to positive selection, which gives the potential for improvement of viral replicative capacity and may lead to more severe disease.

Reviewer #2: Professor van Riel and colleagues report findings from in vitro and in vivo studies that examined the contribution of specific amino acid residues of an H5N1 influenza A virus strain to CNS invasion and neurological disease. Cell culture studies revealed that the CNS-associated mutations enhanced the in vitro viral replication phenotype. Follow-up analysis with the ferret model of influenza and an H5N1 influenza virus strain harboring these putative CNS-associated mutations revealed an attenuated viral phenotype, including restricted tissue distribution. The results suggest the consequence of positive selection on viral phenotypes and potential evolution of influenza viruses.

**Part II – Major Issues: Key Experiments Required for Acceptance**
Please use this section to detail the key new experiments or modifications of existing experiments that should be absolutely required to validate study conclusions.

Generally, there should be no more than 3 such required experiments or major modifications for a "Major Revision" recommendation. If more than 3 experiments are necessary to validate the study conclusions, then you are encouraged to recommend "Reject".

Reviewer #1: 1.Lines 104-111. Please explain why the change in the surface potential from negative to neutral in the apo form and from moderately negative to moderately positive charge in the promotor-bound form influence interactions with potential (host) binging factors?

We thank the reviewer for this suggestion. We have added the following sentences (Line 100-103): “*The surface electrostatic potential is important for the affinity and specificity of macromolecular interactions, protein folding, and chemical reactivity. [15] For example, the adaptive E627K mutation in the PB2 subunit of the RNA polymerase changes the surface potential and influences the interaction with host factor ANP32A or importins-α isoforms. [16-20]*.

2. Lines 133-137. Both the single substitution PB1-652T and the combined substitutions PB1-652T/177G with NP-119M can result in a significant increase of polymerase activity in A549 cells. However, the combination of PB1-652T with NP-119M maintained wild-type levels of polymerase activity. Please give some reasonable explanation for these phenotypes that seem contradictory.

We agree with the reviewer that this might seem contradictory. However, single substitutions can by themselves increase polymerase activity, such as the PB1-652T mutation but also mutations such as the well-known E627K in the PB2 protein. In addition, multiple substitutions can work synergistically (in the case of PB1-652T/177G with NP-119M) whereas combination of one or two of these substitutions individually might not work synergistically and therefor do not result in increased polymerase activity.

Reviewer #2: Genetic analysis of the viral population within the nasal cavity did not reveal a potential bottleneck or selection of virus with CNS-associated mutations. These results suggest the possibility that viral quasispecies contribute to the observed CNS invasive phenotype, neurological disease, and broad tissue distribution, which was lost by selection of a specific viral strain with the CNS-associated mutations.

We thank the reviewer for raising the possibility that the diversity of the viral population itself might modulate phenotypes. However, we currently do not have evidence for this as both the H5N1-CBS and H5N1-WT are generated using reverse genetics, and thus have a low diversity.

Another limitation of the study is that this phenotype was observed for one infected ferret. It is unclear how the host-specific response of this one animal contributed to the observed phenotype and if the selective forces are reproducible in shaping evolution of influenza viruses.

The point of the reviewer is valid in the sense that the CNS-associated mutations were detected in only one animal presenting severe neurological signs. It is therefore unclear whether these specific mutations can be expected to arise independently under similar conditions. However, the aim of the study was to evaluate the adaptive potential of HPAI H5N1 viruses to the CNS. For this reason we choose a ferret in which virus spread efficiently throughout the CNS. In order to make this more clear we have re-written the aim (Line 63-65) as follows “*In the present study, we evaluated an H5N1 HPAI virus that acquired three substitutions – PB1 E177G and A652T and NP I119M - in the CNS of an experimentally inoculated ferret in order to evaluate the adaptive potential of H5N1 virus to the CNS.*

The role of the host-specific responses are indeed unclear, but based on the regular clinical evaluations and the histopathological evaluation at the end of the experiments there was no evidence for underlying conditions that might have influenced the response to the infection..”

**Part III – Minor Issues: Editorial and Data Presentation Modifications**
Please use this section for editorial suggestions as well as relatively minor modifications of existing data that would enhance clarity.

Reviewer #1: 1.It is better to specify which amino acids were site-directed mutated to generate the H5N1-CNS virus in Materials and methods or anywhere proper.

This has been adjusted in the Materials and methods as follows (Line 316-320): “*Substitutions of interest (PB1-E177G, PB1-A652T, NP-I119M) were introduced by site-directed mutagenesis using the QuikChange multi-site-directed mutagenesis kit (Stratagene, Leusden, Netherlands) according to the instructions of the manufacturer to generate the H5N1 viruses with single, double or triple mutants. The virus containing all three variants will be referred to as H5N1-CNS virus.”*

2.Line 125. Where are the large positively charged RNA-biding grove? Please highlight it in the supplementary figure 5.

Supplementary figure 5 has been updated and now includes a highlighted positively charged RNA-biding groove.

3. Line 127. “different ‘human’ cell types” is suggested for more specific description.

Line 127 has been changed accordingly and now reads (Line 126) “CNS-associated mutations enhance polymerase activity in different human cell types.”

4.Fig 5. Whether the difference of genetic diversity is significant statistically?

We did not perform statistical analyses on these data as these are single measurements on individual animals. However, the same trend is observed in all 4 ferrets in which these analyses could be performed.

5.Line 205. The study aimed to explore the CNS-associated mutations of an H5N1 HPAIV, did not involve reassortment events among different viruses, so “variant” may be appropriate than “genotypes”.

This has been adjusted.

6.Lines 222, 223. Please provide more information to explain what the sentence “it is highly unlikely for common variants penetrated the CNS from the nasal turbinate” .

We want to bring to the attention of the reviewer that the sentence reads: “Since it is highly unlikely for common variants at comparable frequencies to arise independently, these data suggest that multiple variants penetrated the CNS from the nasal turbinate”. This means that we found minor variants, at very similar frequencies in the nasal turbinates and different parts of the CNS. We are stating that the possibility of parallel evolution is extremely low compared to an invasion mediated by a rather loose bottleneck. For example, in figure 7, ferret 7 (F7), the blue diamonds and the orange triangle are present in the NT and OB at very similar frequencies. Similarly, OB and BS from ferret 8 show shared minor variants (in blue and green).

7.Line 278. what’s means of “stochastic fixation”？

We here suggest that the initially PB1 E177G was fixed in viruses in the CNS by a chance event, and that PB1 A652T was selected afterwards. To make this clear, we have adjusted the sentence as follows (Line 270-273): “*The increased in vitro polymerase activity implies that the CNS-associated mutations may have been fixed by positive selection but the fact that, in vivo, PB1 E177G reached fixation first while PB1 A652T seemed to offer greater advantage is possibly the result of the stochastic initial fixation of PB1 E177G and posterior selection of PB1 A652T.”*

8. Statistical method in Fig. 2. Whether one-way ANOVA should be used instead of two-way ANOVA?

We thank the reviewer for his observation. Indeed, in Figure 2, panel A contains a typo hat indicated a two-way ANOVA which is incorrect. The statistical analysis in panel A was a one-way ANOVA comparing 1 variable (the polymerase activity of a specific substitution at 12 hours post transfection). The statistical analysis of panel B was indeed a two-way ANOVA which has two variables (a H5N1-WT virus versus the mutant virus H5N1-CNS over time). We have adjusted the figure legend which now reads: “*Mutant PB1 or NP genes that have significantly different polymerase or replicative (B) activity from that of H5N1-WTt are indicated by asterisks identified using a one-way (A) analysis of variance (ANOVA) with Bonferroni’s multiple-comparison tests or a two-way (B) analysis of variance (ANOVA) with Dunnett’s multiple-comparison tests”*

Reviewer #2: not-applicable
